# Effect of combining mosquito repellent and insecticide treated net on malaria prevalence in Southern Ethiopia: a cluster-randomised trial

**DOI:** 10.1186/1756-3305-7-132

**Published:** 2014-03-28

**Authors:** Wakgari Deressa, Yemane Y Yihdego, Zelalem Kebede, Esey Batisso, Agonafer Tekalegne, Getachew A Dagne

**Affiliations:** 1 Department of Preventive Medicine, School of Public Health, Addis Ababa University, Addis Ababa, Ethiopia; 2 Abt Associates, Addis Ababa, Ethiopia; 3 Malaria Consortium Ethiopia, Addis Ababa, Ethiopia; 4 Department of Epidemiology and Biostatistics, College of Public Health, University of South Florida, Tampa FL, USA

## Abstract

**Background:**

A mosquito repellent has the potential to prevent malaria infection, but there has been few studies demonstrating the effectiveness of combining this strategy with the highly effective long-lasting insecticidal nets (LLINs). This study aimed to determine the effect of combining community-based mosquito repellent with LLINs in the reduction of malaria.

**Methods:**

A community-based clustered-randomised trial was conducted in 16 rural villages with 1,235 households in southern Ethiopia between September and December of 2008. The villages were randomly assigned to intervention (mosquito repellent and LLINs, eight villages) and control (LLINs alone, eight villages) groups. Households in the intervention villages received mosquito repellent (i.e., Buzz-Off® petroleum jelly, essential oil blend) applied every evening. The baseline survey was followed by two follow-up surveys, at one month interval. The primary outcome was detection of *Plasmodium falciparum*, *Plasmodium vivax*, or both parasites, through microscopic examination of blood slides. Analysis was by intention to treat. Baseline imbalances and clustering at individual, household and village levels were adjusted using a generalized linear mixed model.

**Results:**

3,078 individuals in intervention and 3,004 in control group were enrolled into the study. Compared with the control arm, the combined use of mosquito repellent and LLINs significantly reduced malaria infection of all types over time [adjusted Odds Ratio (aOR) = 0.66; 95% CI = 0.45-0.97]. Similarly, a substantial reduction in *P. falciparum* malaria infection during the follow-up surveys was observed in the intervention group (aOR = 0.53, 95% CI = 0.31-0.89). The protective efficacy of using mosquito repellent and LLINs against malaria infection of both *P. falciparum/P. vivax* and *P. falciparum* was 34% and 47%, respectively.

**Conclusions:**

Daily application of mosquito repellent during the evening followed by the use of LLINs during bedtime at community level has significantly reduced malaria infection. The finding has strong implication particularly in areas where malaria vectors feed mainly in the evening before bedtime.

**Trial registration:**

ClinicalTrials.gov identifier: NCT01160809.

## Background

Malaria is one of the most important causes of morbidity and mortality in Ethiopia. Annually, about three million cases and 1,700 deaths due to malaria are reported mainly from basic health facilities [1]. Malaria transmission is unstable with marked geographical or seasonal variations [2]. As a result, large-scale epidemics frequently occur [3-5]. *Plasmodium falciparum* and *Plasmodium vivax* are the most prevalent malaria parasites in Ethiopia [5,6] and *Anopheles arabiensis* is the most important malaria vector.

A significant scale-up of insecticide-treated nets (ITNs), particularly the long-lasting insecticidal nets (LLINs), was implemented throughout Ethiopia since 2007 [7,8]. Deployment of this intervention has had a major impact on malaria during the past decade of the Roll Back Malaria (RBM) [9] and reinvigorated the real hope of malaria elimination [10-12]. Ethiopia has adopted the global malaria elimination campaign, as clearly stipulated in its current national strategic plan for malaria prevention and control [13].

The effectiveness of ITNs/LLINs against malaria, however, depends on their acceptability, operational feasibility and proper utilization [14,15]. One of the limitations of the use of mosquito nets is their application only during bedtime. The feeding behaviour of malaria vectors has an important implication in the use of ITNs/LLINs. Mosquitoes can bite between dusk and bedtime while people are engaged in different activities both outdoors and indoors. A study revealed that the peak biting hours for the primary malaria vector in Ethiopia was from 18:00–20:00 (biting indoor) and 22:00–24:00 (biting outdoor) [16]. The time was identified as peak biting hours of mosquitoes before most local people go to bed, suggesting a need to consider additional malaria prevention options.

Studies have shown that mosquito repellents provide protection against malaria infection when used alone compared with a placebo group [17-19]. In Pakistan, where the local vectors start to bite shortly after dusk, the use of di-ethyl 3-methylbenzamid (DEET) mosquito repellent was associated with a 56% reduction in malaria infection [20]. Both mosquito repellents and LLINs have been proven to protect against malaria. Despite the advantages and disadvantages of each of the interventions, it is hypothesized that the use of mosquito repellent during evening can improve the effectiveness of LLINs in preventing malaria. A cluster-randomised controlled trial conducted in the Bolivian Amazon Region showed the effectiveness of combining a plant-based insect repellent with ITNs in reducing malaria infection [21]. This study demonstrated a highly significant reduction in malaria episodes particularly due to *P. vivax* in those who used a combination of treated nets and mosquito repellent. Other studies also suggested the importance of combining mosquito repellents with non-pyrethroid insecticides to improve the efficacy of treated nets against malaria vectors [22-24] and topical repellents for protection against insect bites [25].

Although the major burden of malaria falls in sub-Saharan Africa, no study has been conducted to assess the effect of combining mosquito repellent and LLINs against malaria prevention in the continent. The current study presents findings on the effect of community level combination of mosquito repellent with LLINs against malaria in southern Ethiopia. The aim of this study was to determine whether combining mosquito repellent and LLINs is more effective in reducing malaria prevalence at the community level than using LLINs alone. The primary outcome was detection of malaria parasites (*P. falciparum* plus *P. vivax*) through microscopic examination of blood slides collected from finger-pricks. The findings of this study are expected to be helpful in generating evidence to inform the public about mosquito repellent as protection against malaria and to design an appropriate integrated vector control strategy in the country.

## Methods

### Study area and population

This study was done between September and December 2008 in the rural communities of Halaba woreda (or district) in Southern Nations, Nationalities and People’s Region (SNNPR) of Ethiopia. Located at about 310 km south of Addis Ababa with an area of 91,000 hectares, Halaba woreda is situated between Shashemene and Wolayita Sodo towns. A woreda is further divided into *kebeles*, the smallest local government structure subdivided into *sub-kebeles* (villages). A *kebele* usually consists of about 3000 individuals with around 600 households. According to the 2007 national population census of Ethiopia [26], the population size of the district was 232,223 living in 77 rural and two urban *kebeles*. Children under the age of five years constitute about 18.1% of the total population in the district. The communities of the district are mainly Halaba ethnic in composition, primarily speaking *Halabigna* language. The livelihood of the majority of the inhabitants is mainly based on subsistence farming complemented by livestock rearing. Maize and pepper are the main crops grown in the district.

Formal health services are highly limited in the area. In 2008/09, there were four health centers (two standard and two nucleus health centers) and 57 *kebele*-based health posts in the district. The *kebele*-based health posts are staffed by two female health extension workers (HEWs) and are responsible for about 5,000 people in their respective catchments. Situated at about 1600-1800 m above sea level, the climatic condition of the district is favorable for seasonal mosquito proliferation and malaria transmission. Malaria is the main cause of morbidity and mortality in the district and the transmission exhibits bimodal pattern. The major period of malaria transmission occurs from September to December, following the main rainy season from June to August. The minor peak of transmission occurs from April to May, after a shorter and more erratic March and April rains.

### Sample size calculation

The study was designed as a community-based cluster randomized controlled trial with a primarily quantitative data collection method. This study was based on two population groups: 1) a group of households that use only LLINs (control) and 2) a group of households that use both mosquito repellent and LLINs (intervention). A study conducted in 2007 estimated a malaria prevalence of 5.4% for SNNPR [27]. Based on this prevalence, the sample size of 3,150 in each arm was estimated to provide 80% power to detect a 40% reduction in the prevalence of malaria in the intervention group (P_1_ = 3.25%) compared with a control (P_2_ = 5.4%) at 5% level of significance with 5% non-response rate and a design effect of 2. Assuming an average of five people per household, 630 households were required for each group of the study on the ratio of 1:1 basis. The required number of clusters for analyzing changes in the primary outcome at a minimum of eight villages per arm with 75 households per village was calculated using methods described by Hayes and Bennett [28], with an inter-cluster correlation coefficient of 0.25. For logistical and financial reasons, we did not revise our sample size calculation based on malaria prevalence at baseline.

### Sampling and randomization

Cluster was the unit of randomization. *Kebeles* and villages were randomly chosen at the first and second stages of the sampling, respectively. Due to logistical and inaccessibility problems with a four-wheel drive vehicle, 22 *kebeles* within an hour drive from the woreda capital were eligible for inclusion, of which eight were randomly selected. Each *kebele* was subdivided into villages with an average of about 75 households each. Finally, two villages from each *kebele* were randomly selected and assigned to either the intervention or control group using a lottery method. Randomisation of villages was done by the research team in the presence of independent malaria experts from the District Health Office (DHO). Due to the nature of the intervention, masking of the community and the laboratory technicians who collected blood slides was impossible. However, all the slide readers were blinded to the two arms of the study and to the results of the diagnosis of the preceding readers.

### Baseline and intervention procedures

A baseline census (survey 0), in which all household heads were asked the name, age, and sex of each household member, was carried out in October 2008. Data were collected using pre-tested structured household and individual questionnaires using local language. A baseline survey also established the coverage of malaria control interventions and malaria prevalence. All households in the intervention and control villages were assessed for possession of LLINs. The second survey (survey 1), which was carried out in November 2008, was followed by the third survey (survey 2) just after a month. Blood samples from finger-prick were collected during the follow-up surveys for microscopic detection of the primary outcome variable (*P. falciparum/P. vivax*).

Households with inadequate number, poor status of LLINs or without LLINs were provided with at least one free LLIN after education and demonstration about its use in collaboration with personnel from the DHO. The distribution of LLINs to the households was based on the family size, assuming one LLIN for two people. PermaNet 2.0 LLINs for this purpose were obtained from the Vestergard Frandsen Ethiopia through Coalition Against Malaria in Ethiopia (CAME) and Malaria Consortium Ethiopia. The households in the intervention villages were provided with a mosquito repellent (i.e., Buzz-Off® petroleum jelly and essential oil blend) obtained from the GREEN PLC in Addis Ababa, Ethiopia. It is a jelly applied every evening to exposed areas of the body (face, neck, hands and legs), and one application provides protection against mosquito bite for about eight hours. The GREEN PLC currently produces the product in Addis Ababa. The repellent is prepared in a plastic cup (50 g) that would last an individual for about four weeks of nightly use. The repellent was tested using the WHO standards, and is currently widely used by travelers in Ethiopia and beyond.

One data collector and additional intervention educator (local community resident) were recruited from each village and received three days intensive training. The intervention educators actively engaged in mobilization of the community. Field educators educated and demonstrated all household members on the application of mosquito repellent. Each household member including children above one year old was intended to apply the repellent. Further education and demonstration coupled with monitoring and supervision for compliance of the repellent continued for the whole intervention period. The intervention educators in the intervention villages promoted the proper use of both the repellent and LLINs for the community, while these activities to promote community engagement and participation were only limited to the use of LLINs in the control villages.

### Blood slide collection and laboratory analysis

Experienced laboratory technicians prepared thick and thin blood films from finger-prick blood samples on a slide from all consenting individuals. The films were labeled and air-dried horizontally in a slide tray in the field. Households with absent individuals were revisited on the same or the following day for blood sample collection. Using the standard malaria laboratory procedures, the thin films of the blood slides were fixed with methanol, and the thin and thick films stained with 3% Giemsa for about 10–20 minutes. A senior malaria laboratory expert examined the blood slides for malaria parasites using Olympus light microscope. Blood slides were declared negative based on the examination of 100 high power microscopic fields under oil immersion. A second reading for all positive slides and a random sample of 5% of the negative slides was performed by a highly experienced malaria laboratory microscopist. Any discordant results of the blood slides between the first and the second reader was resolved by a qualified laboratory technologist.

### Data analysis

Data were entered using Epi Info version 6.04d, and analysed with STATA version 10 and SAS version 9.3 (SAS 9.3). We analyzed the data to examine the effect of mosquito repellent and LLINs on our primary outcome. No adjustments were made for missing individuals and we did intention to treat analysis, including all people who were enrolled and given finger-prick samples for smear preparation. Malaria prevalence was estimated for each of the three surveys. Differences in proportions were compared for significance using X^2^ tests, with p value of less than 0.05. The 95% confidence intervals were calculated and presented for most of the point estimates. A logistic mixed effects model using SAS 9.3 was carried out to assess the effect of mosquito repellent and LLIN, compared with the LLIN alone group, on malaria prevalence and infection with falciparum malaria, moderated by time of survey. The analysis included the following independent variables: time of survey, gender, age, type of intervention, interaction between time and intervention status, baseline household ownership of LLIN and IRS status. Interaction was assessed between the intervention and time in the final model. Clustering at village, household and individual levels were accounted for by the logistic mixed effects model. The model also accounts for baseline differences in malaria prevalence between the study arms.

### Ethical considerations

This study received ethical approval and clearance from the Institutional Review Board of SNNPR Health Bureau, and the Ministry of Ethiopian Science and Technology. The study population included all members of households in the selected villages. Local community leaders, elders and all heads of households were informed about the study to gain support for the project. Verbal informed consent was obtained from each adult. For children under 18 years of age, consent was provided by the parents or guardians. Blood samples through finger-pricks were collected aseptically using disposable lancets, and treatment was provided according to the national guideline for malaria cases microscopically identified [29]. Confidentiality was ensured at all levels of the study. LLINs were freely distributed at baseline to households in both intervention and control villages. At the end of the study, one bottle of mosquito repellent was distributed to households in the control group after giving education on its use and demonstration of its application on the skin. This study is registered with ClinicalTrials.gov, number NCT01160809.

### Role of the funding source

The sponsor of the study and the suppliers of the repellent and LLINs had no role in the study design, data collection, analysis, interpretation, dissemination, writing of the manuscript or in the decision to submit this paper for publication. The corresponding author had full access to all the data in the study and had final responsibility for the decision to submit for publication.

## Results

### Baseline characteristics

6,080 people from 1,235 households [615 (49.8%) intervention and 620 (50.2%) control] participated in the study. The number of people and households per study village was well balanced between the intervention and control groups. At baseline (survey 0), 2,622 (85.2%) of 3,078 individuals in the intervention and 2600 (86.6%) of 3004 people in the control were tested for malaria parasites. Table 1 shows the baseline demographic and socio-economic characteristics of the study households by study arm. There was no statistically significant difference between intervention and control groups in the average size of households, proportions of children less than five years old and pregnant women, and household’s proxy indicators of wealth. However, ownership of nets by households and use by children younger than five years and pregnant women were higher in the control than the intervention group.

**Table 1:** Baseline demographic and socio-economic characteristics of the study participants by study arm

**Baseline characteristics**	**Study arm**		**Total**
	**Repellent and LLINs**	**LLINs alone**	
Number of households enrolled, n (%)	615 (49.8)	620 (50.2)	1235 (100)
Total household members enrolled, n (%)	3088 (50.6)	3004 (49.4)	6082 (100)
Average (SD) household size	5.0 (2.0)	4.8 (1.9)	4.9 (2.0)
Mean (SD) age (years)	19.0 (14.6)	19.6 (15.4)	19.3 (15.0)
Female (%)	49.5	50.7	50.1
Children <5 years (%)	15.4	15.5	15.5
Pregnant women (%)	1.42	1.30	1.4
Households with:			
Corrugated iron roof (%)	4.4	2.9	3.6
Pipe water supply (%)	38.5	25.8	32.1
Pit latrine (%)	74.0	84.4	79.2
At least one ox (%)	70.3	67.9	69.3
Bicycle (%)	15.4	12.9	14.2
At least one LLIN (%)	56.6	65.6	61.1
Two or more LLIN (%)	14.8	35.8	25.3
People slept under an LLIN during the night preceding the survey (%)	41.2	58.9	50.5
Children <5 years slept under an LLIN during the night preceding the survey (%)	55.7	75.7	66.6
Pregnant women slept under an LLIN during the night preceding the survey (%)	40.0	71.4	60.0

LLIN = Long-lasting insecticidal net, SD = Standard deviation.

Of 1,235 households, household ownership of at least one LLIN was 61.1% (95% CI = 58-64) (56.6% in repellent vs. 65.6% in control group); 38.4% of households had no net of any type at the time of the baseline survey (Table 1). Households in the control group were more likely to own one or more LLINs than those in the repellent group, and marked differences in possession of two or more LLINs particularly existed between the intervention (14.7%, 95% CI = 12.1-17.9) and control (35.8%, 95% CI = 32.0-39.7) groups at baseline (p < 0.01). The percentage of people including children the age of five and pregnant women who slept under an LLIN from the control households was higher than those from the intervention households. The imbalance in the ownership of LLINs by the households in both arms of the study was successfully corrected with free supply of LLINs at baseline. The malaria parasite prevalence by blood slide microscopy at baseline is also shown in Table 1, with marked differences between the repellent (2.5%, 95% CI = 1.9-3.1) and the control (1.2%, 95% CI = 0.8-1.7, p < 0.01) groups. An adjustment of the baseline imbalance of malaria prevalence between the two groups was made during statistical analysis.

### Study participant flow

**Figure 1:**
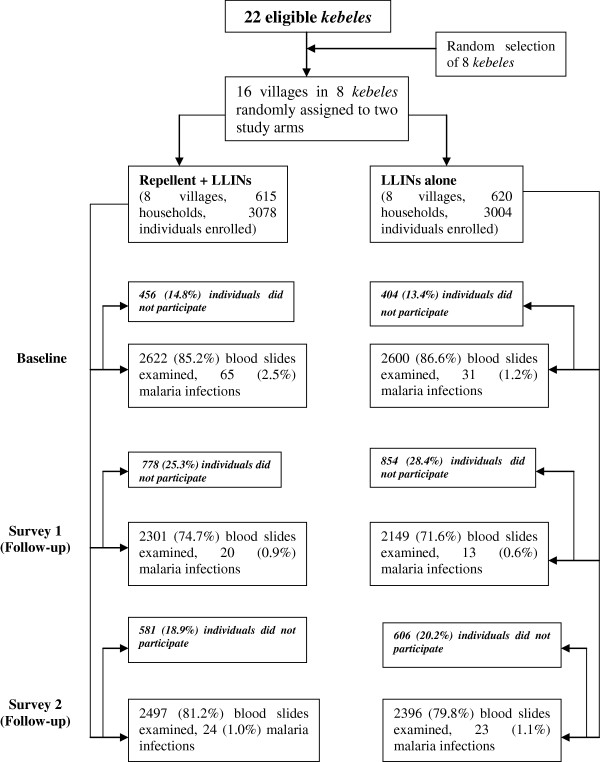
Trial profile.

Figure 1 shows the trial profile. At baseline (survey 0), 2,622 (85.2%) of 3,078 enrolled individuals in the intervention and 2,600 (86.6%) of 3,004 enrolled people in the control group were tested for malaria parasites. The numbers of people who failed to give finger-prick for blood slide at baseline were similar in the intervention and control group (14.8% and 13.8%, respectively). At follow-up during survey 1, 74.7% of the initially enrolled individuals in the intervention and 71.6% of those in the control group were examined for malaria parasites. During the third survey (survey 2), 81.2% of individuals in the intervention and 79.8% in the control group were examined for malaria blood slide microscopy.

### Malaria between intervention and control groups

**Figure 2:**
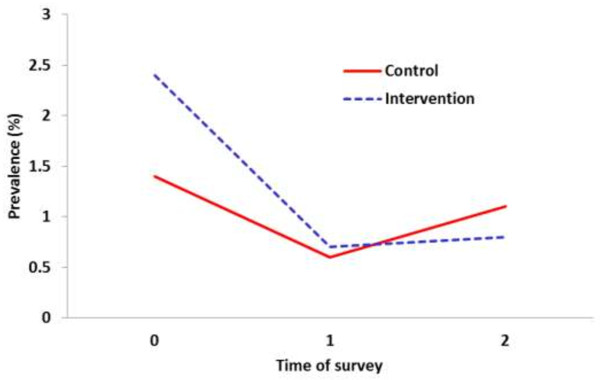
Trends in the unadjusted reduction of the prevalence of malaria infection between the two groups.

A total of 14,565 (80%) finger-prick blood slides were obtained from 18,244 expected individuals during the baseline and the two follow-up surveys, of which 176 (1.2%, 95% CI = 1.0-1.4) were positive for malaria parasites (58% *P. falciparum* and 41.5% *P. vivax*) (Table 2). At baseline, 1.8% (95% CI = 1.5-2.2) of the obtained smears were positive for malaria infections, with marked differences between the intervention (2.5%, 95% CI = 1.9-3.1) and the control (1.2%, 95% CI = 0.8-1.7) groups. The overall unadjusted trend of the effect of the combined use of mosquito repellent and LLINs shows that malaria infection in the intervention was dramatically reduced and became almost equal to the prevalence in the control group at survey 1 (Figure 2). The reduction in malaria in the repellent and LLINs group was also pronounced during the last survey and reduced to a level lower than in the control, but the prevalence in the latter group was raised.

**Table 2:** Number of blood slides examined and malaria prevalence by study arm in the baseline and follow-up surveys

**Study arm**	**Number (%)**		
	**Baseline**	**Survey 1**	**Survey 2**
***Repellent + LLINs group***			
People in the households	3078	3079	3078
Finger-prick blood slides examined	2622 (85.2)	2301 (74.7)	2497 (81.1)
Slides positive for any malaria parasite	65 (2.5)	20 (0.9)	24 (1.0)
*P. falciparum* only	42 (1.6)	14 (0.6)	9 (0.4)
*P. vivax* only	23 (0.9)	6 (0.3)	15 (0.6)
Mixed infections (*Pf/Pv*)	0 (0.0)	0 (0.0)	0 (0.0)
***LLINs alone group***			
People in the households	3004	3003	3002
Finger-prick blood slides examined	2600 (86.6)	2149 (71.6)	2396 (79.8)
Slides positive for any malaria parasite	31 (1.2)	13 (0.6)	23 (1.0)
*P. falciparum* only	18 (0.7)	6 (0.3)	13 (0.5)
*P. vivax* only	12 (0.5)	7 (0.3)	10 (0.4)
Mixed infections (*Pf/Pv*)	1 (0.04)	0 (0.0)	0 (0.0)
***Total***			
People in the households	6082	6082	6080
Finger-prick blood slides examined	5222 (85.9)	4450 (73.2)	4893 (80.5)
Slides positive for any malaria parasite	96 (1.8)	33 (0.7)	47 (1.0)
*P. falciparum* only	60 (1.1)	20 (0.4)	22 (0.4)
*P. vivax* only	35 (0.7)	13 (0.3)	25 (0.5)
Mixed infections (*Pf/Pv*)	1 (0.02)	0 (0.0)	0 (0.0)

LLIN = Long-lasting insecticidal net, *P. f* = *Plasmodium falciparum; P. v* = *Plasmodium vivax.*

Due to the differences in the baseline, a logistic mixed effects model analysis was used to determine the effect of mosquito repellent and LLIN (compared with the LLIN group only) on malaria (a dichotomous outcome variable) over a period of two months with two follow-up measurements and baseline. Table 3 presents the outputs of the logistic mixed effects regression analysis using odds ratio, including the effects of other covariates such as time of survey, sex (male/female), age (under five, 5 to 14, and ≥15 years), repellent and LLINs vs. control group, household’s ownership of at least one LLIN at baseline (yes/no), and IRS status of the study villages (yes/no). The findings indicate that the use of mosquito repellent with LLINs is found to be highly effective in reducing malaria infection in comparison to the control group (LLIN alone).

**Table 3:** Results of logistic mixed effects model analysis to compare malaria infection between the intervention (repellent and LLIN) and control (LLIN only)

**Variables**	**Adjusted odds ratio (OR)**	**95% Confidence interval for OR**	**P-value**
Sex (male)	0.80	0.57-1.13	0.201
Age group			
Under 5	3.55	2.40-5.27	<0.0001
5-14	2.30	1.58-3.36	0.0002
15 and above	1.00		
Time	0.87	0.65-1.16	0.335
Repellent and LLIN (yes)	1.99	1.07-3.71	0.049
Time* repellent and LLIN	**0.66**	**0.45-0.97**	**0.033**
Baseline household LLIN ownership (yes)	0.76	0.53-1.09	0.141
IRS status (yes)	1.26	0.68-2.35	0.474

LLIN = Long lasting insecticidal net, IRS = Indoor residual spraying, OR = Odds Ratio, *Interaction term (Time and repellent/LLIN).

**Figure 3:**
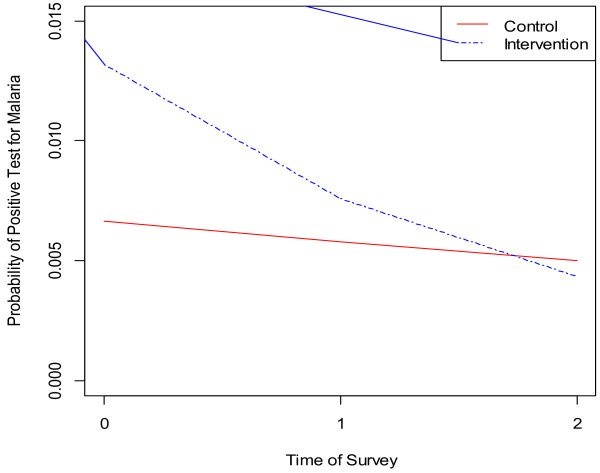
Probability of malaria positive for intervention conditions over time.

Calculated with the logistic mixed effects regression model, the effect of a repellent and LLIN expressed as an adjusted odds ratio (OR) is 0.66 (95% CI = 0.46-0.97). Moderated by time of survey (follow-up), the protective efficacy of mosquito repellent and LLIN against malaria compared with LLIN alone group is 34%. As compared to individuals who were older than 15 years, younger children had a significantly higher chance of malaria infection (OR = 3.55, 95% CI: 2.40-5.27). The effect of the intervention gets stronger over time after controlling for the covariates (Figure 3). Over time, the probability of a positive test for malaria declines faster for the intervention group than the control group.

**Figure 4:**
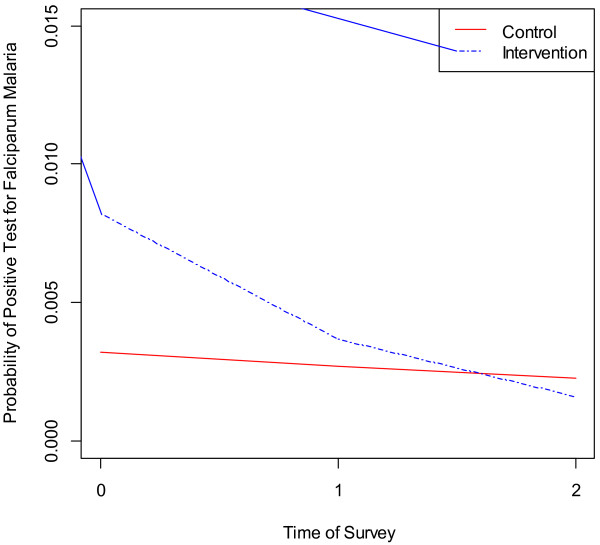
Probability of falciparum malaria positive for intervention conditions over time.

The logistic mixed effects model analysis was also extended to determine the effect of the combined use of mosquito repellent and LLIN on *P. falciparum* infection only (Table 4). The results demonstrate a statistically significant effect of the intervention on falciparum malaria infections over time (adjusted OR = 0.53, 95% CI = 0.31-0.89; p = 0.017). Protective efficacy of repellent and LLINs against falciparum malaria infection was 47%. Over time, the probability of a positive test for *P. falciparum* declines faster for intervention group than that of the control group after controlling for covariates (Figure 4). No statistically significant reduction with *P. vivax* infection was noted between the intervention and control arms of the study.

**Table 4:** **Results of logistic mixed effects model analysis to compare *P. falciparum *** **malaria infection between the intervention (repellent and LLIN) and control (LLIN only)**

**Variables**	**Adjusted odds ratio (OR)**	**95% Confidence interval for OR**	**P-value**
Sex (male)	0.81	0.51-1.27	0.3550
Age group			
Under 5	3.07	1.82-5.16	0.0002
5-14	2.20	1.36-3.57	0.0032
15 and above	1.00		
Time	0.84	0.57-1.24	0.3830
Repellent and LLIN (yes)	2.59	1.24-5.40	0.0250
Time* repellent and LLIN	**0.53**	**0.31-0.89**	**0.0170**
Baseline household LLIN ownership (yes)	1.77	0.88-3.53	0.1317
IRS status (yes)	0.77	0.48-1.24	0.2860

LLIN = Long lasting insecticidal net, IRS = Indoor residual spraying, OR = Odds Ratio, *Interaction term (Time and repellent/LLIN).

## Discussion

The study findings show that combining mosquito repellent with LLINs markedly reduced the prevalence of malaria in southern Ethiopia. The results also show a more beneficial effect of the intervention on the prevalence of *P. falciparum* malaria. Although 72-87% of the total individuals provided blood samples during the baseline and follow-up surveys, there were no marked differences in the background characteristics of the study participants between the study groups except the baseline imbalances in the ownership of LLINs and the prevalence of malaria, which were adjusted during the analysis, suggesting that the effects of the intervention were not affected by selection bias. However, selection biases still may result because inaccessible and faraway *kebeles* were excluded from the sampling frame.

The study area is characterized by unstable and seasonal malaria transmission. The prevalence of malaria at baseline was 1.8%, higher than the 0.6% of the national malaria indicator survey (MIS) 2007 report for SNNPR [7], but lower than the 2.5% prevalence estimated by the MIS 2011 for the region [8]. The prevalence of falciparum malaria in the SNNPR increased from 0.2% in 2007 [7] to 2.2% in 2011 [8]. However, the overall malaria incidence in Ethiopia has shown a dramatic decline between 2005 and 2010, attributed mainly to the massive scale up control interventions [30,31]. Although malaria transmission is seasonal in the study area, there is no evidence that this introduced bias on the effect of the intervention on the prevalence of malaria since conditions were similar in both arms of the study from initial baseline to follow-up levels.

The study findings show a reduction in the prevalence of malaria over time in the intervention compared with the control, after adjusting for baseline differences and other covariates. This was associated with approximately a 34% reduction in the odds of all malaria infection and a 50% reduction in falciparum malaria during the follow-up surveys in the intervention group. It should be noted that the achieved reduction in malaria infection in the intervention group to the level lower than the control was achieved from a statistically significant differences at baseline characteristics. This suggests that a significant number of infective bites occur in the evening before bedtime. Moreover, the repellent is also probably providing protection for most part of the night for people sleeping outside nets for various reasons. In fact, the combination of mosquito repellent with LLINs may be particularly effective in Ethiopia and similar settings where the primary malaria vectors such as *An. arabiensis* and *An. gambiae s.s.* are mostly anthropophagic [16]. In fact, many recent studies suggest the combined strategy of vector control interventions against malaria infection despite a considerable debate about the relative added value of each of the interventions [32-34].

This study does not indicate a significant reduction in the prevalence of *P. vivax* infections between the intervention and control groups. Although studies from the Bolivian Amazon [21] and eastern Afghanistan [19] showed a pronounced effect of combining repellent with ITNs on the prevalence of malaria due to *P. vivax*, other studies showed no significant effect of repellent against *P. vivax* malaria [20]. Although difficult to explain, the most likely explanation was because of the relapses of infections before the trial or the small number of *P. vivax* infections occurred during the study. In Ethiopia, radical cure for malaria due to *P. vivax* infections using primaquine is not recommended in malarious areas due to reinfection problems.

This study has some limitations. Differences in the main outcome variable (malaria infection) and household ownership of LLIN which could have some confounding effects were accounted for in multivariate regression models. The study was based only on malaria infections detected through the surveys and did not take into account malaria infections developed, detected and treated through passive surveillance between the surveys. The study had only two arms and the effect of a third arm with only mosquito repellent has not been conducted as the use of LLINs is an established national intervention. The possibility of contamination is plausible because the study villages were geographically contiguous, with some degree of movement and communication among villages. Even if this study was conducted during peak malaria transmission season, the prevalence of malaria was lower, and it does not represent the effect of the interventions on malaria throughout the year. Although the coverage of the intervention households with mosquito repellent was universal, the daily application of the repellent by every household member was difficult to ensure, leading to compliance problems.

Despite the above limitations this study certainly provides important evidence on the benefits of combining mosquito repellent with LLINs at community level for malaria prevention. Although the major burden of malaria falls in sub-Saharan Africa, there is little evidence on the complementary effects of the two interventions against malaria in the continent. Most people often stay outdoors during the evening and go to bed late in the night, exposing themselves to early biting mosquitoes. This necessitates the need to search for other protection methods before people go to bed and use nets. The protective efficacy of our findings was substantial, and the use of mosquito repellent at the community level should be explored. However, these findings should be confirmed with a larger sample size in similar or different epidemiological settings with strong passive surveillance and active surveillance across different seasons. In addition, a cost-effectiveness analysis of the combination of the two interventions should be assessed to determine the best use of the meager resources since their implementation demands additional resources.

## Conclusion

This study demonstrates that daily application of mosquito repellent during the evening followed by the use of LLINs during bedtime at the community level has significantly reduced malaria infection. This finding suggests that the use of mosquito repellent during the evening can improve the effectiveness of LLINs against malaria and has important implications for malaria control programmes particularly in areas where vectors feed mainly in the evening. In addition, repellents could also help control outdoor biting vectors.

## Competing interests

The authors declare that they have no competing interests.

## Authors’ contributions

WD involved in proposal writing, designed the study and participated in coordination, supervision and the overall implementation of the project. He analysed the data, drafted and finalized the manuscript. YYY came up with the concept, designed the study, and reviewed the manuscript. ZK involved in the study design, supervision and coordination of field work, and reviewed the manuscript. EB involved in coordination and supervision of the field work, and reviewed the manuscript. AT received funding for the study and participated in all stages of the project implementation and revision of the manuscript. GAD did all the advanced statistical analysis and critical revision of the paper for intellectual content. All authors read and approved the final version of the manuscript.
